# Supplementary material for: Molecular Phylogenetics of Seven Cyprinidae Distant Hybrid Lineages: Genetic Variation, 2nNCRC Convergent Evolution, and Germplasm Implications
Source: Biology (Basel). 2025 Oct 30;14(11):1527. doi: 10.3390/biology14111527 (PMC12650161; doi:10.3390/biology14111527)
Supplement: Supplementary file 1 [file biology-14-01527-s001.zip › Figure S1.pdf]

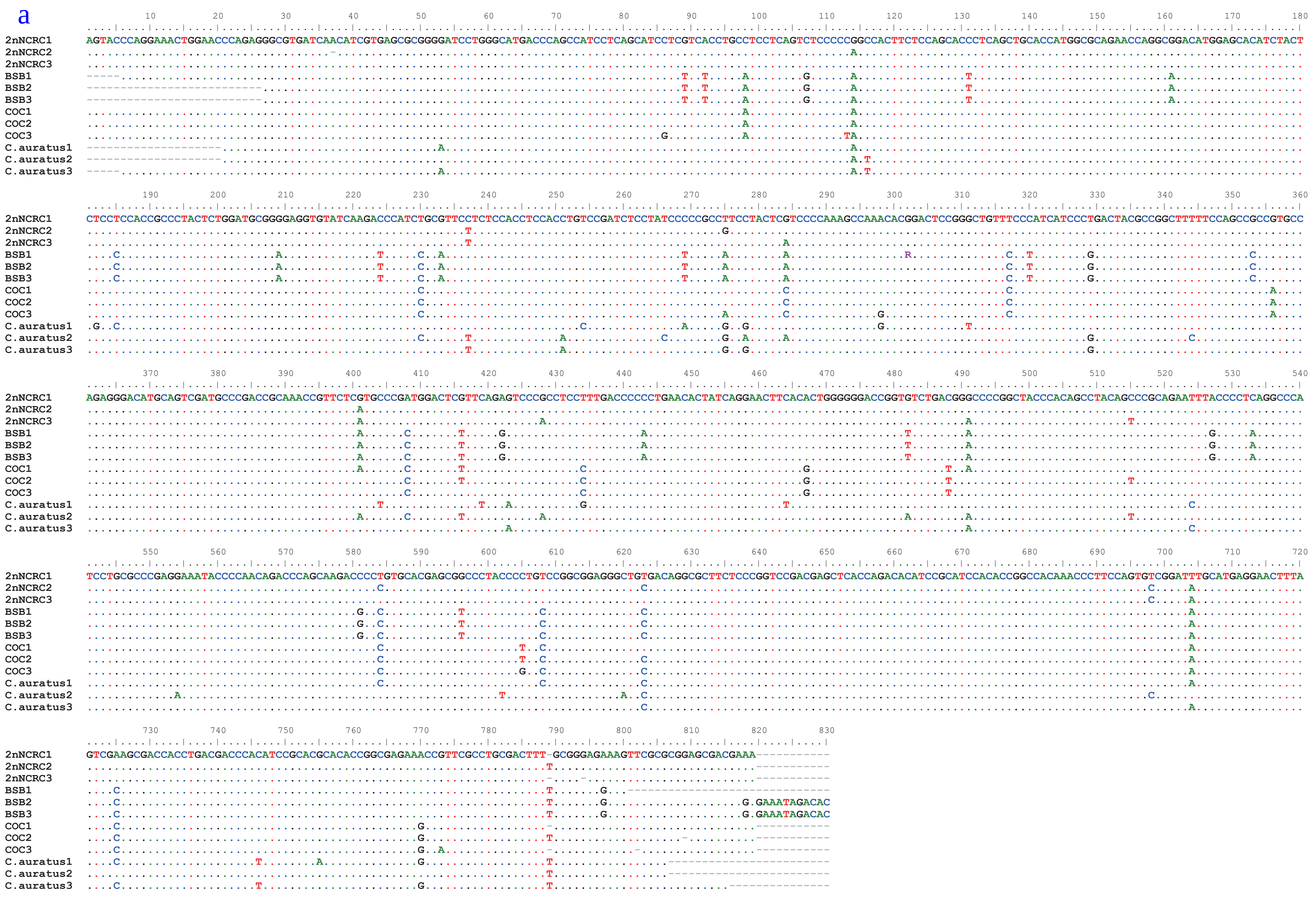
a
2nNCRC1
2nNCRC2
2nNCRC3
BSB1
BSB2
BSB3
COC1
COC2
COC3
C.auratus1
C.auratus2
C.auratus3

b

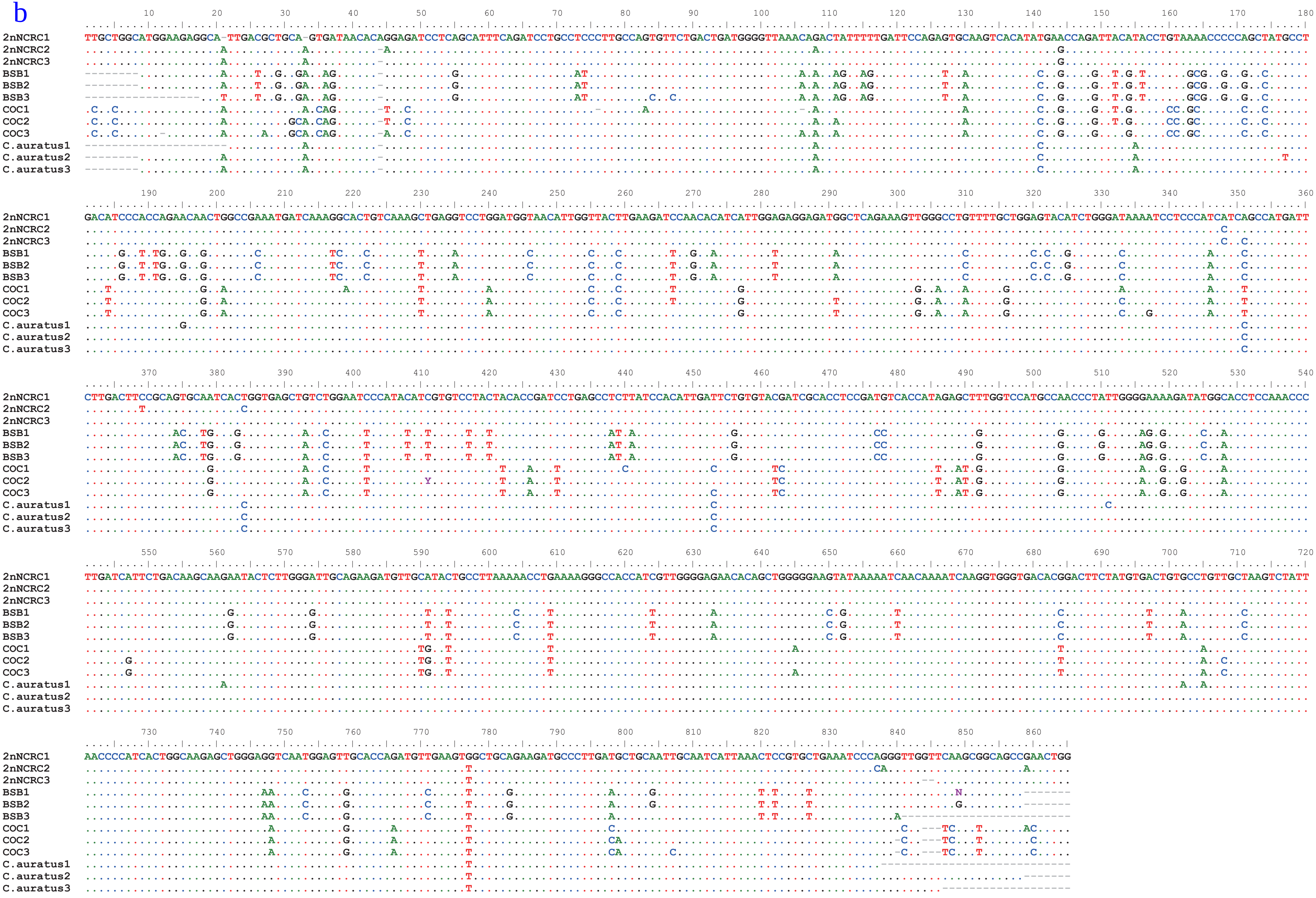
2nNCRC1
2nNCRC2
2nNCRC3
BSB1
BSB2
BSB3
COC1
COC2
COC3
C.auratus1
C.auratus2
C.auratus3

C

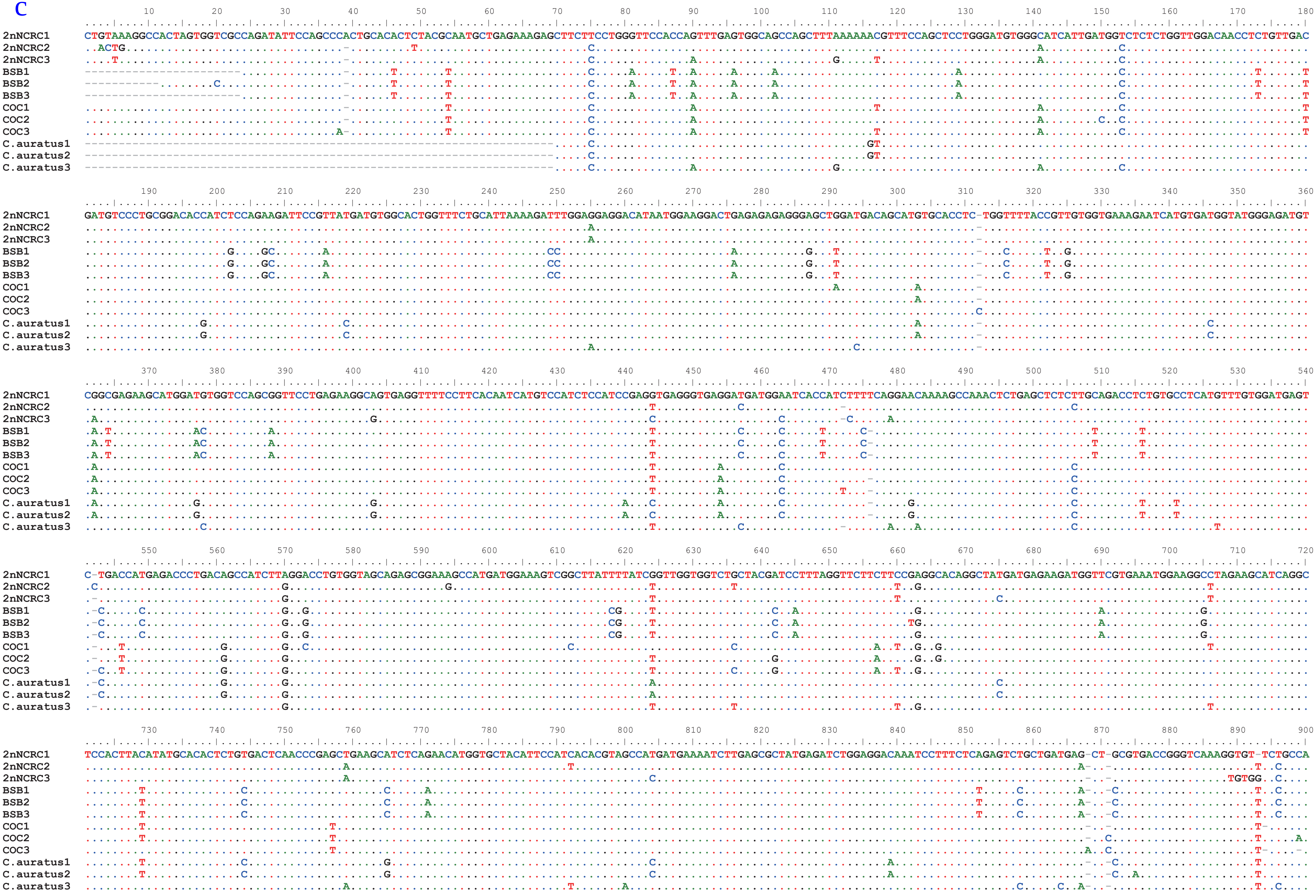

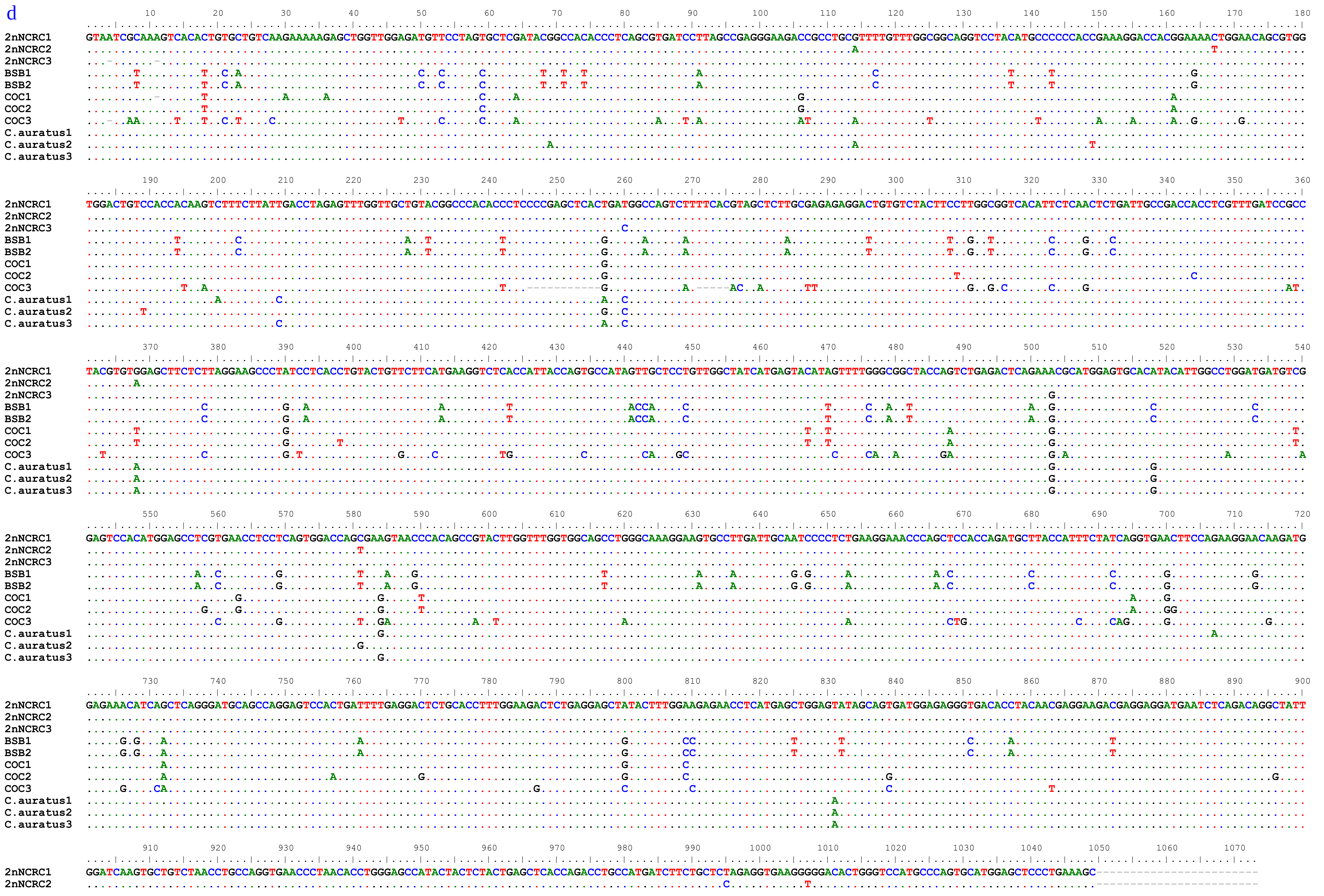
d
2nNCRC1
2nNCRC2
2nNCRC3
BSB1
BSB2
COC1
COC2
COC3
C.auratus1
C.auratus2
C.auratus3

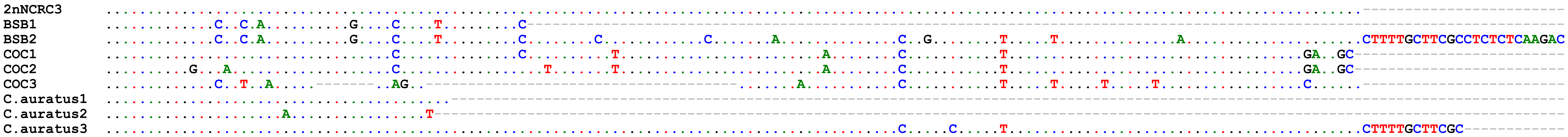
2nNCRC3
BSB1
BSB2
COC1
COC2
COC3
C.auratus1
C.auratus2
C.auratus3

e

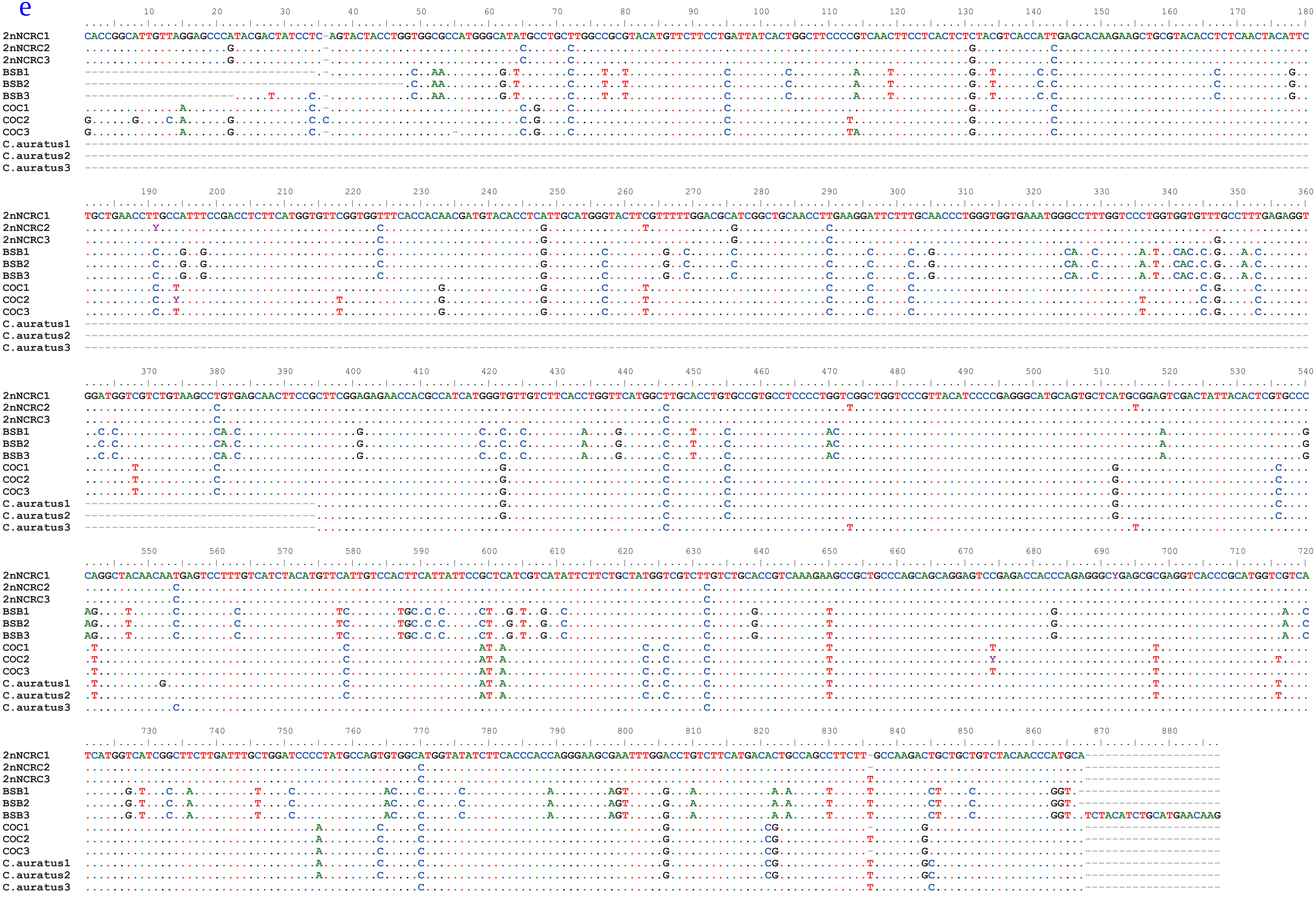
2nNCRC1
2nNCRC2
2nNCRC3
BSB1
BSB2
BSB3
COC1
COC2
COC3
C.auratus1
C.auratus2
C.auratus3

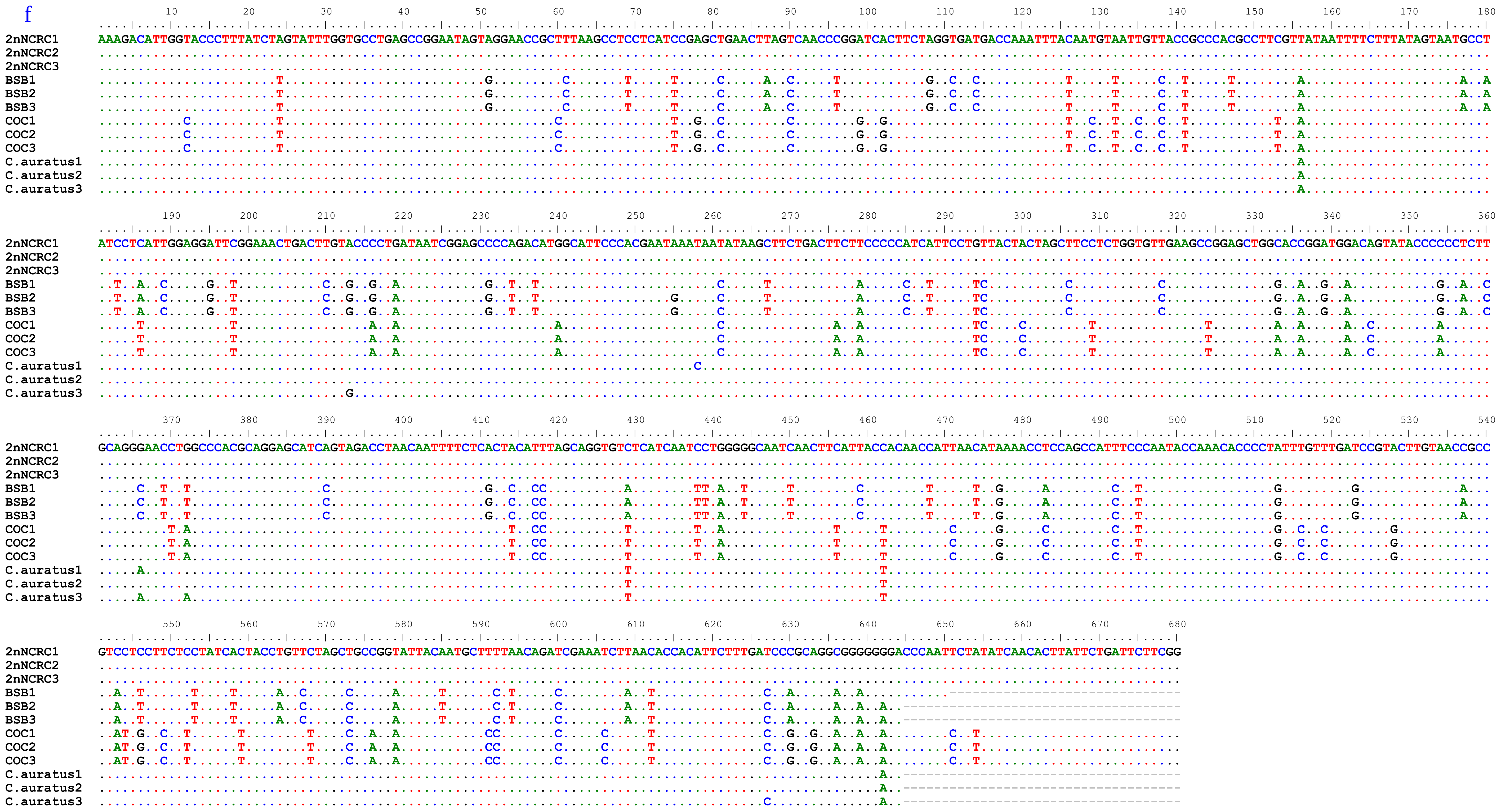
f
2nNCRC1
2nNCRC2
2nNCRC3
BSB1
BSB2
BSB3
COC1
COC2
COC3
C.auratus1
C.auratus2
C.auratus3

g

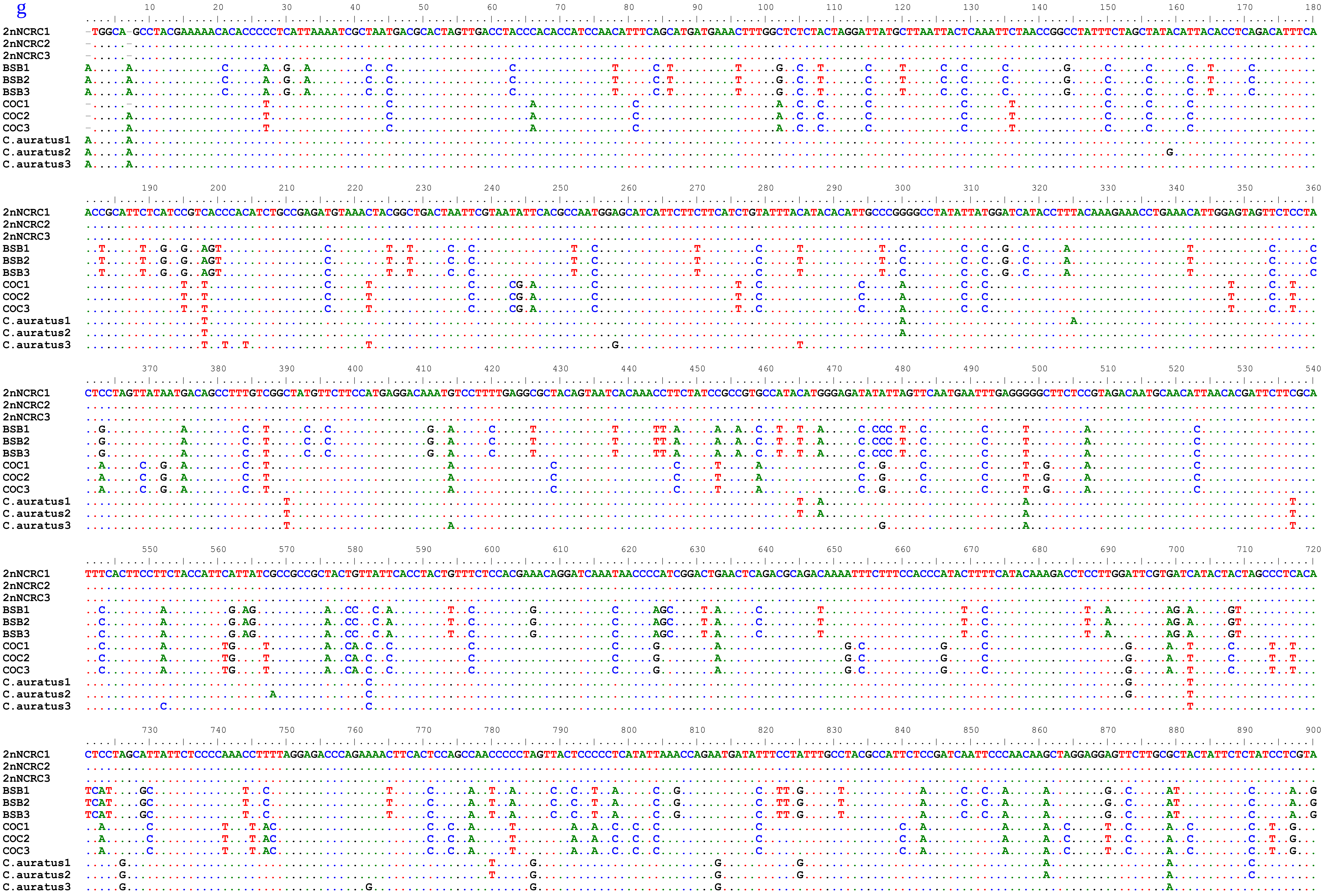
2nNCRC1
2nNCRC2
2nNCRC3
BSB1
BSB2
BSB3
COC1
COC2
COC3
C.auratus1
C.auratus2
C.auratus3

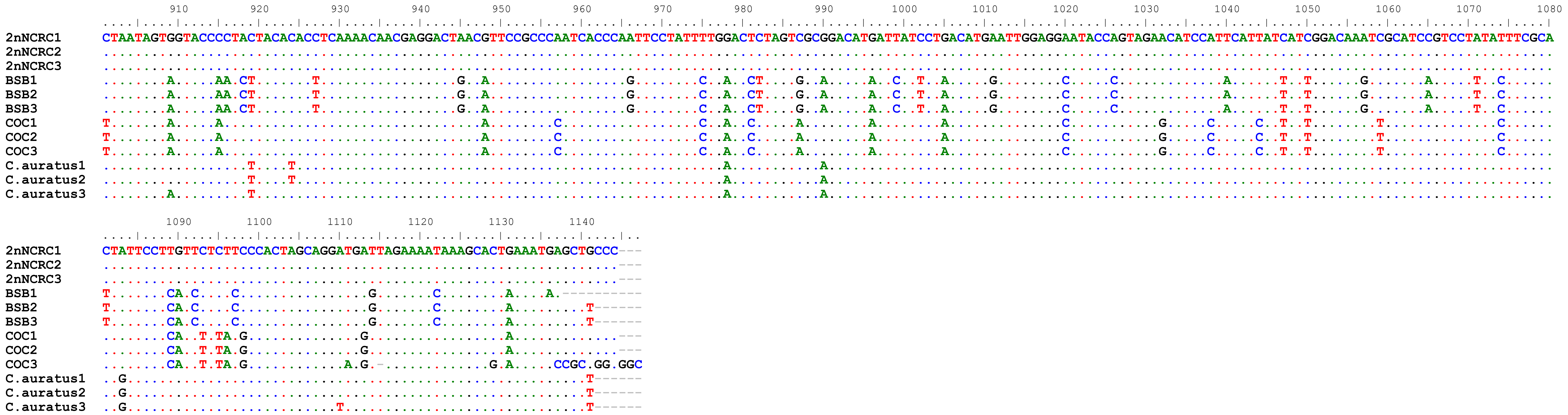
910 920 930 940 950 960 970 980 990 1000 1010 1020 1030 1040 1050 1060 1070 1080
2nNCRC1 CTAATAGTGGTACCCCTACTACACACCTCAAAACAACGAGGACTAACGTTCCGCCCAATCACCCAATTCCTATTTTGGACTCTAGTCGCGGACATGATTATCCTGACATGAATTGGAGGAATACCAGTAGAACATCCATTCATTATCATCGGACAAATCGCATCCGTCCTATATTTCGCA
2nNCRC2
2nNCRC3
BSB1
BSB2
BSB3
COC1
COC2
COC3
C.auratus1
C.auratus2
C.auratus3
1090 1100 1110 1120 1130 1140
2nNCRC1 CTATTCCTTGTTCTCTTCCCACTAGCAGGATGATTAGAAAATAAAGCACTGAAATGAGCTGCCC
2nNCRC2
2nNCRC3
BSB1
BSB2
BSB3
COC1
COC2
COC3
C.auratus1
C.auratus2
C.auratus3

h

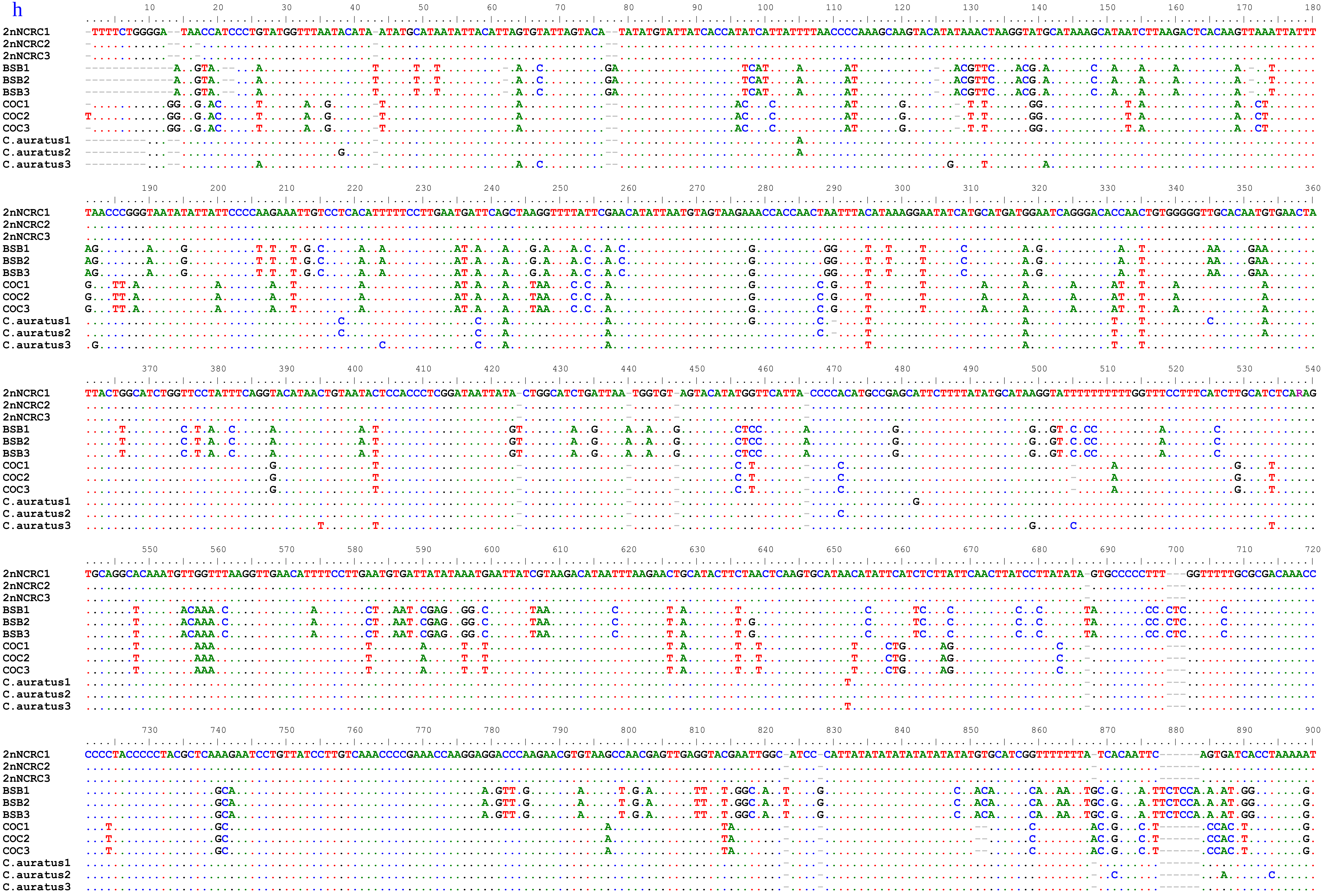
2nNCRC1
2nNCRC2
2nNCRC3
BSB1
BSB2
BSB3
COC1
COC2
COC3
C.auratus1
C.auratus2
C.auratus3

```
                 910       920       930       940       950       960
             ....|....|....|....|....|....|....|....|....|....|....|....|.
2nNCRC1      CTCTGCCAAAAACCCCAAAAAATCACCTCCACACTAAATTTTCTAACAT-ATTAGCT----
2nNCRC2      .................................................-..-....----
2nNCRC3      .........................................-.......-.......----
BSB1         .C..ATT...--TTTTT.GGGCG.G..C.A.TG.....AAG..C.....T.AA.AGC----
BSB2         .C..ATT...--TTTTT.GGGCG.G..C.A.TG.....AAG..C.....T.AA.AGC----
BSB3         .C..ATT...--TTTTT.GGGCG.G..C.A.TG.....AAG..C.....T.AA.AGC----
COC1         .C..A.......T...C.....GAGG...GG........AC.....T..A..-.ATCAGCT
COC2         .C..A.......T...C.....GAGG...GG........AC.....T..A....ATCAGCT
COC3         .C..A.......T...C.....GAGG...GG........AC.....T..A....ATCAGCT
C.auratus1   .................................................T...TA------
C.auratus2   .................................................T...TA------
C.auratus3   .................................................T...TA------
```

i

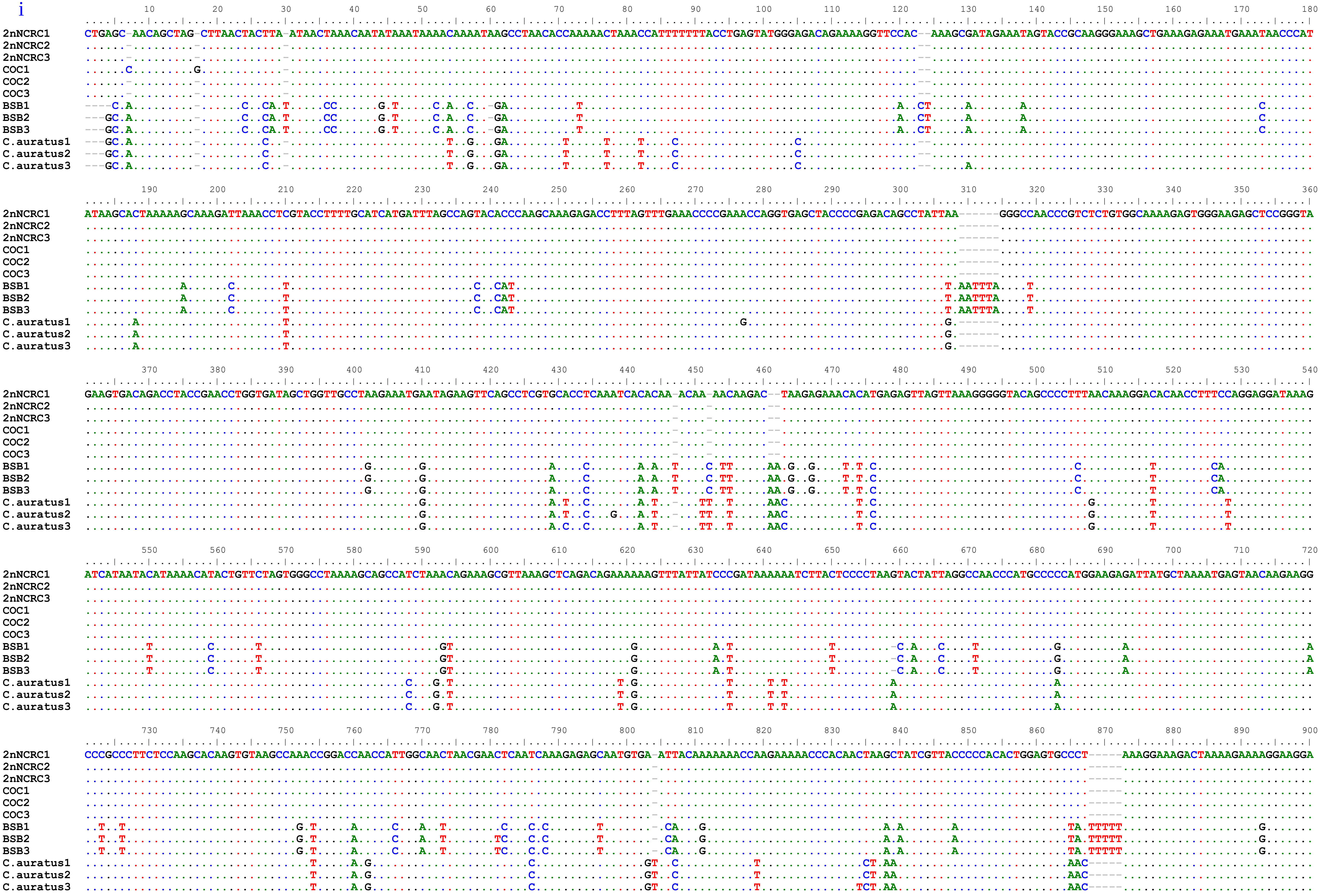
2nNCRC1
2nNCRC2
2nNCRC3
COC1
COC2
COC3
BSB1
BSB2
BSB3
C.auratus1
C.auratus2
C.auratus3

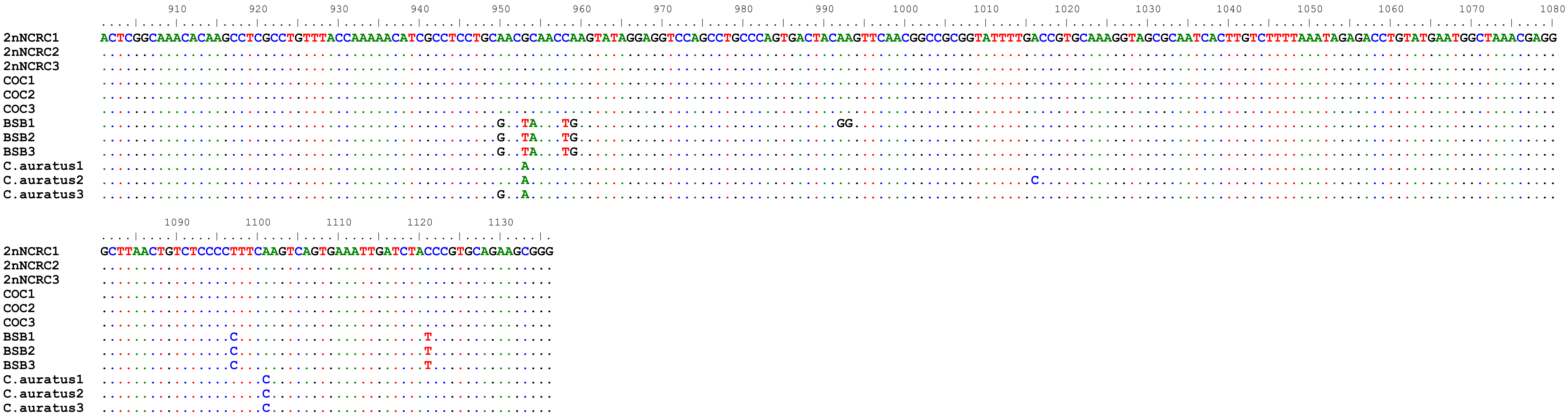

Figure S1. The alignment of the nucleotide sequences of (a) *EGR2b*, (b) *IRBP2*, (c) *Rag1*, (d) *Rag2*, (e) *RH2*, (f) *COI*, (g) *Cytb*, (h) *D-loop*, (i) *16S rRNA* among the distant hybrids 2nNCRC, parents (COC♀, BSB♂), and wild crucian carp (*C. auratus*)
