# Supplementary material for: Molecular Phylogenetics of Seven Cyprinidae Distant Hybrid Lineages: Genetic Variation, 2nNCRC Convergent Evolution, and Germplasm Implications
Source: Biology (Basel). 2025 Oct 30;14(11):1527. doi: 10.3390/biology14111527 (PMC12650161; doi:10.3390/biology14111527)
Supplement: Supplementary file 1 [file biology-14-01527-s001.zip › Figure S2.pdf]

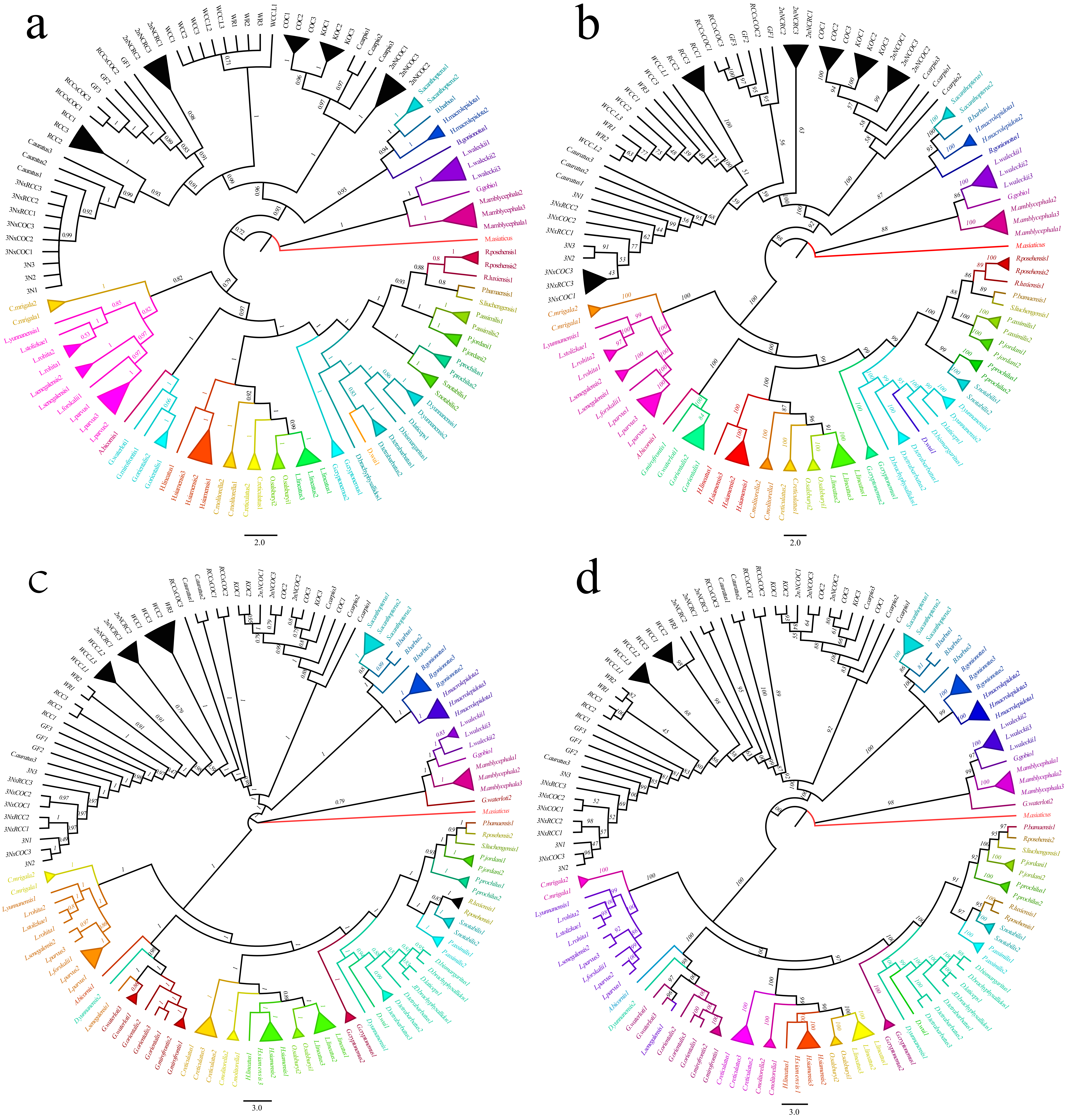

Figure S2. Phylogenies reconstructed from concatenated mitochondrial (mtDNA) and nuclear (nDNA) gene datasets, (a) Bayesian mtDNA tree; (b) ML mtDNA tree; (c) Bayesian nDNA tree; (d) ML nDNA tree.
