# Supplementary material for: Molecular Phylogenetics of Seven Cyprinidae Distant Hybrid Lineages: Genetic Variation, 2nNCRC Convergent Evolution, and Germplasm Implications
Source: Biology (Basel). 2025 Oct 30;14(11):1527. doi: 10.3390/biology14111527 (PMC12650161; doi:10.3390/biology14111527)
Supplement: Supplementary file 1 [file biology-14-01527-s001.zip › Table S11.pdf]

Table S11. Calculating genetic distance in *RH2* sequences within each distant hybrid strain, as well as within each Cyprinidae species based on the Kimura 2-Parameter model.

**Species**	**Genetic distance**
C.carpio	0.0036
C.auratus	0.0366
H.macrolepidota	0.0000
B.gonionotus	0.0017
B.barbus	0.0000
P.assimilis	0.0066
D.yunnanensis	0.0634
P.jordani	0.0066
O.salsburyi	0.0044
C.molitorella	0.0115
P.prochilus	0.0066
G.orientalis	0.0000
D.tetrabarbatus	0.0118
R.posehensis	0.0385
S.notabilis	0.0044
L.senegalensis	0.0755
L.lineatus	0.0113
L.rohita	0.0000
H.siamensis	0.0033
C.reticulatus	0.0000
C.mrigala	0.0025
L.parvus	0.0000
L.waleckii	0.0000
BSB	0.0000
COC	0.0070
KOC	0.0050
WCC-L	0.0117
GF	0.0148
RCCxCOC	0.0137
2nNCRC	0.0144
2nNCOC	0.0093
3NxCOC	0.0101
3NxRCC	0.0050
3N	0.0077
WR	0.0070
WCC	0.0164
RCC	0.0140
