# Supplementary material for: Molecular Phylogenetics of Seven Cyprinidae Distant Hybrid Lineages: Genetic Variation, 2nNCRC Convergent Evolution, and Germplasm Implications
Source: Biology (Basel). 2025 Oct 30;14(11):1527. doi: 10.3390/biology14111527 (PMC12650161; doi:10.3390/biology14111527)
Supplement: Supplementary file 1 [file biology-14-01527-s001.zip › Table S2-3.pdf]

**Table S2.** Comparative analysis of transition/transversion ratio (R), indel variations, and synonymous/nonsynonymous substitution rate (Ks) per gene in distant hybrids and parents.

| Genes | Distant hybrids | Parents | | Ks (mean ± SD) | Indels | R |
|---|---|---|---|---|---|---|
| *16S rRNA* | 2*n*NCOC | COC | ♀ | 0.0513±0.0000 | 3 | 2.3 |
| | | BSB | ♂ | 0.0903±0.0005 | 22 | 1.5 |
| | 2*n*NCRC | COC | ♀ | 0.0000 | 0 | 2.1 |
| | | BSB | ♂ | 0.0842±0.0005 | 24 | n/c |
| | 3N×RCC | 3N | ♀ | 0.0003±0.0004 | 1 | 0.0 |
| | | RCC | ♂ | 0.0054±0.0019 | 0 | 1.9 |
| | 3N×COC | 3N | ♀ | 0.0000 | 1 | n/c |
| | | COC | ♂ | 0.0492±0.0000 | 4 | 2.3 |
| | RCC×COC | RCC | ♀ | 0.0072±0.0019 | 1 | 1.9 |
| | | COC | ♂ | 0.0521±0.0000 | 4 | 2.3 |
| | WCC-L | WCC | ♀ | 0.0000 | 1 | n/c |
| | | BSB | ♂ | 0.0945±0.0005 | 21 | 1.8 |
| | WR | WCC | ♀ | 0.0003±0.0004 | 2 | 0.0 |
| | | RCC | ♂ | 0.0272±0.0023 | 2 | 3.5 |
| *COI* | 2*n*NCOC | COC | ♀ | 0.0015±0.0000 | 0 | n/c |
| | | BSB | ♂ | 0.1438±0.0000 | 0 | 2.3 |
| | 2*n*NCRC | COC | ♀ | 0.1228±0.0000 | 0 | 1.7 |
| | | BSB | ♂ | 0.1534±0.0017 | 0 | 2.9 |
| | 3N×RCC | 3N | ♀ | 0.0000 | 0 | n/c |
| | | RCC | ♂ | 0.0054±0.0007 | 0 | n/c |
| | 3N×COC | 3N | ♀ | 0.0000 | 0 | n/c |
| | | COC | ♂ | 0.1176±0.0000 | 0 | 3.1 |
| | RCC×COC | RCC | ♀ | 0.0094±0.0007 | 0 | 2.3 |
| | | COC | ♂ | 0.1228±0.0000 | 0 | 2.9 |
| | WCC-L | WCC | ♀ | 0.0005±0.0007 | 0 | 0.0 |
| | | BSB | ♂ | 0.1686±0.0017 | 0 | 2.0 |
| | WR | WCC | ♀ | 0.0005±0.0007 | 0 | 0.0 |
| | | RCC | ♂ | 0.0491±0.0008 | 0 | 9.9 |
| *Cytb* | 2*n*NCOC | COC | ♀ | 0.0047±0.0017 | 1 | 0.4 |
| | | BSB | ♂ | 0.1659±0.0015 | 2 | 1.8 |
| | 2*n*NCRC | COC | ♀ | 0.1244±0.0015 | 1 | 2.3 |
| | | BSB | ♂ | 0.1844±0.0005 | 5 | 2.6 |
| | 3N×RCC | 3N | ♀ | 0.0000 | 1 | n/c |
| | | RCC | ♂ | 0.0141±0.0000 | 1 | n/c |
| | 3N×COC | 3N | ♀ | 0.0000 | 0 | n/c |
| | | COC | ♂ | 0.1295±0.0039 | 4 | 2.7 |
| | RCC×COC | RCC | ♀ | 0.0223±0.0000 | 0 | n/c |
| | | COC | ♂ | 0.1258±0.0029 | 2 | 2.5 |
| | WCC-L | WCC | ♀ | 0.0003±0.0004 | 2 | 0.0 |
| | | BSB | ♂ | 0.1789±0.0007 | 7 | 2.1 |
| | WR | WCC | ♀ | 0.0003±0.0004 | 0 | 0.0 |

| | | | | | | |
|---|---|---|---|---|---|---|
| | | RCC | ♂ | 0.071±0.0004 | 0 | 16.0 |
| *D-loop* | 2*n*NCOC | COC | ♀ | 0.0055±0.0000 | 1 | n/c |
| | | BSB | ♂ | 0.2384±0.0007 | 45 | 1.5 |
| | 2*n*NCRC | COC | ♀ | 0.1326±0.0000 | 13 | 1.6 |
| | | BSB | ♂ | 0.2397±0.0039 | 43 | 1.4 |
| | 3N×RCC | 3N | ♀ | 0.0000 | 2 | n/c |
| | | RCC | ♂ | 0.02±0.001 | 1 | 8.6 |
| | 3N×COC | 3N | ♀ | 0.0000 | 1 | n/c |
| | | COC | ♂ | 0.1239±0.0000 | 11 | 1.1 |
| | RCC×COC | RCC | ♀ | 0.016±0.0005 | 3 | 7.9 |
| | | COC | ♂ | 0.1304±0.0006 | 14 | 1.3 |
| | WCC-L | WCC | ♀ | 0.0000 | 3 | n/c |
| | | BSB | ♂ | 0.2399±0.0007 | 29 | 1.5 |
| | WR | WCC | ♀ | 0.0163±0.002 | 4 | n/c |
| | | RCC | ♂ | 0.016±0.0028 | 5 | 2.9 |
| *EGR2b* | 2*n*NCOC | COC | ♀ | 0.011±0.002 | 0 | 1.9 |
| | | BSB | ♂ | 0.0426±0.0022 | 25 | 1.5 |
| | 2*n*NCRC | COC | ♀ | 0.0276±0.0033 | 0 | 1.5 |
| | | BSB | ♂ | 0.0468±0.0019 | 37 | 2.5 |
| | 3N×RCC | 3N | ♀ | 0.0094±0.0028 | 1 | 3.4 |
| | | RCC | ♂ | 0.1846±0.0047 | 1 | 4.0 |
| | 3N×COC | 3N | ♀ | 0.0069±0.0022 | 5 | 4.8 |
| | | COC | ♂ | 0.0333±0.0032 | 4 | 2.9 |
| | RCC×COC | RCC | ♀ | 0.0213±0.0036 | 3 | 2.1 |
| | | COC | ♂ | 0.0168±0.003 | 2 | 1.8 |
| | WCC-L | WCC | ♀ | 0.0193±0.0034 | 3 | 2.5 |
| | | BSB | ♂ | 0.045±0.0051 | 30 | 1.5 |
| | WR | WCC | ♀ | 0.0163±0.002 | 2 | 2.7 |
| | | RCC | ♂ | 0.0161±0.0026 | 2 | 2.9 |
| *IRBP2* | 2*n*NCOC | COC | ♀ | 0.0172±0.0028 | 5 | 1.1 |
| | | BSB | ♂ | 0.1389±0.0036 | 16 | 1.4 |
| | 2*n*NCRC | COC | ♀ | 0.0919±0.0029 | 4 | 1.7 |
| | | BSB | ♂ | 0.1308±0.0411 | 15 | 1.7 |
| | 3N×RCC | 3N | ♀ | 0.0047±0.0035 | 6 | 1.2 |
| | | RCC | ♂ | 0.0126±0.0053 | 8 | 1.3 |
| | 3N×COC | 3N | ♀ | 0.002±0.0016 | 3 | n/c |
| | | COC | ♂ | 0.0945±0.0023 | 2 | 1.8 |
| | RCC×COC | RCC | ♀ | 0.0198±0.0045 | 4 | 1.5 |
| | | COC | ♂ | 0.0794±0.0021 | 4 | 1.6 |
| | WCC-L | WCC | ♀ | 0.016±0.0000 | 3 | 1.8 |
| | | BSB | ♂ | 0.1444±0.0014 | 20 | 1.8 |
| | WR | WCC | ♀ | 0.0055±0.004 | 5 | 1.9 |
| | | RCC | ♂ | 0.0073±0.0035 | 3 | 2.0 |
| *Rag1* | 2*n*NCOC | COC | ♀ | 0.0169±0.0029 | 3 | 1.7 |

| | | | | | | |
|---|---|---|---|---|---|---|
| | | BSB | ♂ | 0.0694±0.0024 | 24 | 1.5 |
| | 2*n*NCRC | COC | ♀ | 0.032±0.0024 | 2 | 1.8 |
| | | BSB | ♂ | 0.075±0.0028 | 23 | 2.2 |
| | 3N×RCC | 3N | ♀ | 0.0093±0.0013 | 3 | 1.7 |
| | | RCC | ♂ | 0.0135±0.0033 | 2 | 1.6 |
| | 3N×COC | 3N | ♀ | 0.0105±0.0018 | 2 | 1.7 |
| | | COC | ♂ | 0.035±0.0037 | 6 | 1.7 |
| | RCC×COC | RCC | ♀ | 0.024±0.0024 | 7 | 2.3 |
| | | COC | ♂ | 0.0184±0.0028 | 7 | 2.5 |
| | WCC-L | WCC | ♀ | 0.0145±0.0047 | 8 | 2.5 |
| | | BSB | ♂ | 0.0701±0.0006 | 28 | 1.5 |
| | WR | WCC | ♀ | 0.012±0.0005 | 2 | 1.9 |
| | | RCC | ♂ | 0.0127±0.0018 | 3 | 1.5 |
| *Rag2* | 2*n*NCOC | COC | ♀ | 0.0794±0.0412 | 44 | 1.4 |
| | | BSB | ♂ | 0.1325±0.0046 | 44 | 1.5 |
| | 2*n*NCRC | COC | ♀ | 0.0578±0.0373 | 0 | 1.8 |
| | | BSB | ♂ | 0.0959±0.0012 | 0 | 2.1 |
| | 3N×RCC | 3N | ♀ | 0.001±0.0014 | 1 | n/c |
| | | RCC | ♂ | 0.0138±0.0012 | 1 | 3.6 |
| | 3N×COC | 3N | ♀ | 0.001±0.0014 | 0 | n/c |
| | | COC | ♂ | 0.0581±0.0369 | 1 | 2.1 |
| | RCC×COC | RCC | ♀ | 0.0307±0.0122 | 4 | 1.0 |
| | | COC | ♂ | 0.06±0.0356 | 3 | 1.3 |
| | WCC-L | WCC | ♀ | 0.0067±0.0017 | 0 | 1.6 |
| | | BSB | ♂ | 0.1025±0.0012 | 0 | 1.9 |
| | WR | WCC | ♀ | 0.0107±0.0076 | 4 | 1.7 |
| | | RCC | ♂ | 0.0082±0.0049 | 4 | 1.5 |
| *RH2* | 2*n*NCOC | COC | ♀ | 0.0079±0.0015 | 0 | 6.8 |
| | | BSB | ♂ | 0.139±0.0018 | 37 | 1.2 |
| | 2*n*NCRC | COC | ♀ | 0.0505±0.0015 | 0 | 1.2 |
| | | BSB | ♂ | 0.1405±0.0032 | 36 | 2.2 |
| | 3N×RCC | 3N | ♀ | 0.0088±0.0033 | 4 | 7.0 |
| | | RCC | ♂ | 0.0188±0.0029 | 4 | 6.5 |
| | 3N×COC | 3N | ♀ | 0.0075±0.0024 | 2 | 5.5 |
| | | COC | ♂ | 0.0592±0.0044 | 3 | 2.5 |
| | RCC×COC | RCC | ♀ | 0.0366±0.0032 | 5 | 2.0 |
| | | COC | ♂ | 0.0216±0.0038 | 4 | 1.9 |
| | WCC-L | WCC | ♀ | 0.0145±0.0042 | 3 | 2.4 |
| | | BSB | ♂ | 0.1343±0.0025 | 39 | 1.1 |
| | WR | WCC | ♀ | 0.0131±0.0022 | 1 | 2.0 |
| | | RCC | ♂ | 0.0096±0.0035 | 1 | 3.6 |

Due to division by zero, the result is marked as n/c (not computable).

**Table S3.** Comparative analysis of transition/transversion ratio (R), indel variations, and synonymous/nonsynonymous substitution rate (Ks) per gene in 2*n*NCRC and wild crucian carp (*C. auratus*).

| Genes | | | Ks (mean ± SD) | Indels | R |
|---|---|---|---|---|---|
| *16S rRNA* | 2*n*NCRC | *C. auratus* | 0.0513±0.0008 | 8 | 2.4 |
| *COI* | 2*n*NCRC | *C. auratus* | 0.0119±0.0024 | 0 | 4.9 |
| *Cytb* | 2*n*NCRC | *C. auratus* | 0.0211±0.0004 | 5 | 13.6 |
| *D-loop* | 2*n*NCRC | *C. auratus* | 0.0213±0.0025 | 14 | 5.5 |
| *EGR2b* | 2*n*NCRC | *C. auratus* | 0.0268±0.0063 | 34 | 2.4 |
| *IRBP2* | 2*n*NCRC | *C. auratus* | 0.0120±0.0029 | 27 | 1.7 |
| *Rag1* | 2*n*NCRC | *C. auratus* | 0.0393±0.0109 | 0 | 2.4 |
| *Rag2* | 2*n*NCRC | *C. auratus* | 0.0107±0.0021 | 0 | 2.5 |
| *RH2* | 2*n*NCRC | *C. auratus* | 0.0386±0.0210 | 0 | 2.0 |
