# Supplementary material for: Molecular Phylogenetics of Seven Cyprinidae Distant Hybrid Lineages: Genetic Variation, 2nNCRC Convergent Evolution, and Germplasm Implications
Source: Biology (Basel). 2025 Oct 30;14(11):1527. doi: 10.3390/biology14111527 (PMC12650161; doi:10.3390/biology14111527)
Supplement: Supplementary file 1 [file biology-14-01527-s001.zip › Table S5.pdf]

Table S5. Calculating genetic distance in *COI* sequences within each distant hybrid strain, as well as within each Cyprinidae species based on the Kimura 2-Parameter model.

**Species**	**Genetic distance**
C. carpio	0.0026
C. auratus	0.0074
H. macrolepidota	0.0000
S. acanthopterus	0.0000
P. assimilis	0.0000
D. yunnanensis	0.0000
P. jordani	0.0000
O. salsburyi	0.0000
C. molitorella	0.0000
P. prochilus	0.0000
G. orientalis	0.0000
D. tetrabarbatus	0.0000
R. posehensis	0.0000
S. notabilis	0.0000
L. senegalensis	0.0000
L. lineatus	0.0000
L. rohita	0.0000
H. siamensis	0.0052
C. reticulatus	0.0000
C. mrigala	0.0236
L. parvus	0.0254
L. waleckii	1.2347
M. amblycephala	0.0013
COC	0.0000
KOC	0.0000
WCC-L	0.0000
GF	0.0010
RCC×COC	0.0000
2*n*NCRC	0.0000
2*n*NCOC	0.0000
3N×COC	0.0000
3N×RCC	0.0000
3N	0.0000
WR	0.0000
WCC	0.0010
RCC	0.0010
