# Supplementary material for: Molecular Phylogenetics of Seven Cyprinidae Distant Hybrid Lineages: Genetic Variation, 2nNCRC Convergent Evolution, and Germplasm Implications
Source: Biology (Basel). 2025 Oct 30;14(11):1527. doi: 10.3390/biology14111527 (PMC12650161; doi:10.3390/biology14111527)
Supplement: Supplementary file 1 [file biology-14-01527-s001.zip › Table S7.pdf]

Table S7. Calculating genetic distance in *Cytb* sequences within each distant hybrid strain, as well as within each Cyprinidae species based on the Kimura 2-Parameter model.

**Species**	**Genetic distance**
C.carpio	0.01181
C.auratus	0.01364
H.macrolepidota	0.0000
S.acanthopterus	0.0000
P.assimilis	0.0000
D.yunnanensis	0.0000
P.jordani	0.0000
O.salsburyi	0.0000
C.molitorella	0.0000
P.prochilus	0.0000
G.cryptonemus	0.0018
G.orientalis	0.0000
D.tetrabarbatus	0.0000
R.posehensis	0.0000
S.notabilis	0.0000
L.senegalensis	0.0000
L.lineatus	0.0000
L.rohita	0.0035
H.siamensis	0.0095
C.reticulatus	0.0000
C.mrigala	0.0259
L.parvus	0.0089
L.waleckii	0.0059
BSB	0.0006
COC	0.0024
KOC	0.0023
WCC-L	0.0018
GF	0.0012
RCCxCOC	0.0012
2nNCRC	0.0000
2nNCOC	0.0018
3NxCOC	0.0000
3NxRCC	0.0000
3N	0.0000
WR	0.0006
WCC	0.0000
RCC	0.0000
