# Supplementary material for: Molecular Phylogenetics of Seven Cyprinidae Distant Hybrid Lineages: Genetic Variation, 2nNCRC Convergent Evolution, and Germplasm Implications
Source: Biology (Basel). 2025 Oct 30;14(11):1527. doi: 10.3390/biology14111527 (PMC12650161; doi:10.3390/biology14111527)
Supplement: Supplementary file 1 [file biology-14-01527-s001.zip › Table S9.pdf]

Table S9. Calculating genetic distance in *RAG1* sequences within each distant hybrid strain, as well as within each Cyprinidae species based on the Kimura 2-Parameter model.

**Species**	**Genetic distance**
C.carpio	0.0304
C.auratus	0.0424
H.macrolepidota	0.0006
B.gonionotus	0.0010
B.barbus	0.0124
S.acanthopterus	0.0013
P.assimilis	0.0021
D.yunnanensis	0.0574
P.jordani	0.0014
O.salsburyi	0.0036
C.molitorella	0.0064
P.prochilus	0.0007
G.cryptonemus	0.0014
G.mirofrontis	0.0014
G.orientalis	0.0099
D.tetrabarbatus	0.0055
R.posehensis	0.0112
S.notabilis	0.0133
L.senegalensis	0.0801
L.lineatus	0.0023
L.rohita	0.0000
H.siamensis	0.0000
C.reticulatus	0.0051
G.waterloti	0.0957
L.parvus	0.0063
BSB	0.0009
COC	0.0159
KOC	0.0190
WCC-L	0.0152
GF	0.0125
RCCxCOC	0.0147
2nNCRC	0.0287
2nNCOC	0.0228
3NxCOC	0.0121
3NxRCC	0.0098
3N	0.0113
WR	0.0037
WCC	0.0106
RCC	0.0175
